# GWAS in interaction with childhood traumas implicates novel variants and genes previously associated with suicide-related factors in the background of suicidal ideation

**DOI:** 10.1192/j.eurpsy.2024.695

**Published:** 2024-08-27

**Authors:** X. Gonda, S. Krause, B. Erdelyi-Hamza, S. Sutori, Z. Gal, N. Eszlari, G. Bagdy, G. Juhasz, D. Torok

**Affiliations:** ^1^ NAP3.0-SE Neuropsychopharmacology Research Group- Hungarian Brain Research Program; ^2^Department of Psychiatry and Psychotherapy; ^3^Doctoral School of Mental Health Sciences; ^4^Institute of Pharmacodynamics, Semmelweis University, Budapest, Hungary

## Abstract

**Introduction:**

Although suicide claims more lives than war and homicide, we still have no sufficient and effective methods either for its prediction or for its prevention. Our screening methods are laborous and subjective both on the side of the patient and on the side of the clinician. Understanding the genetic background of suicidal behaviour would help identify biomarkers for screening as well as pathways as potential targets for novel intervention and prevention approaches. However, in spite of a number of GWAS studies, results are few and rarely replicate, and generally accurate phenotyping and sufficient consideration of environmental stressors is also missing.

**Objectives:**

In our present study we performed a genome-wide analysis study for suicidal ideation in interaction with early childhood traumas in a deep-phenotyped general population sample.

**Methods:**

Our analysis used data from 1800 volunteers in the NewMood project. As outcome phenotype the suicidal ideation item of the Brief Symptom Inventory was used. A modified version of the Childhood Trauma Questionnaire was used to assess early adverse experiences. A genome-wide association analysis was performed with Plink 1.9, including a total of 3,474,641 variants after quality control steps, followed by genome-wide by environment interaction analyses. Our models included control variables for sex, age, and the top 10 genomic principal components. Functional annotation of SNPs was carried out using FUMA v1.5.6, gene-based tests were performed using MAGMA v1.08.

**Results:**

7 SNPs met suggestive significance in main effect analyses, of which 2 reached genome-wide significance including *rs79912020* (p=3.21E-10, β=0.746) and *rs10236520* (p=1.71E-08, β=0.484), with no significant findings in gene-based tests. Interaction analyses with childhood adversities yielded 31 SNPs that met genome-wide significance, including *rs7983955* (p=2.28E-11, β=0.182), *rs141039461* (p=3.90E-11, β=0.0541), *rs12692827* (p=3.69E-10, β=0.0612) as the top SNPs. In interaction with childhood adversities, 31 genes showed a significant association in gene-based tests, including *RBFOX1* (p=1.09E-10), *GRM7* (p=1.20E-10), *MTCH1* (p=5.59E-09), and *CDH13* (p=6.60E-09) as the most significant findings.

**Conclusions:**

Our results indicate several important novel SNPs associated with suicidal ideation when considered in interaction with the effect of childhood adversities. Furthermore, gene-based analyses replicate several genes playing a key role in central nervous system function such as *GRM7* (encoding metabotropic glutamate receptor 7) or previously implicated in association with suicide (*CDH13*) or suicide-related factors such as aggression (*RBFOX1*).

Funding: NAP2022-I-4/2022, K143391, 2019-2.1.7-ERA-NET-2020-00005, TKP2021-EGA-25

**Disclosure of Interest:**

None Declared

